# Different neuroprognostication thresholds of neuron-specific enolase in shockable and non-shockable out-of-hospital cardiac arrest: a prospective multicenter observational study in Korea (the KORHN-PRO registry)

**DOI:** 10.1186/s13054-023-04603-6

**Published:** 2023-08-09

**Authors:** Youn-Jung Kim, Yong Hwan Kim, Chun Song Youn, In Soo Cho, Su Jin Kim, Jung Hee Wee, Yoo Seok Park, Joo Suk Oh, Byung Kook Lee, Won Young Kim

**Affiliations:** 1 Department of Emergency Medicine, Asan Medical Center, University of Ulsan College of Medicine, 88, Olympic-ro 43-gil, Songpa-gu, Seoul, 05505 Korea; 2 Departments of Emergency Medicine, Samsung Changwon Hospital, Sungkyunkwan University School of Medicine, Changwon, Korea; 3 Department of Emergency Medicine, Seoul St. Mary’s Hospital, College of Medicine, The Catholic University of Korea, Seoul, Korea; 4 Department of Emergency Medicine, Hanil General Hospital, Seoul, Korea; 5 Department of Emergency Medicine, Korea University College of Medicine, Seoul, Korea; 6 Department of Emergency Medicine, Yeouido St. Mary’s Hospital, The Catholic University of Korea College of Medicine, Seoul, Korea; 7 Department of Emergency Medicine, Yonsei University College of Medicine, Seoul, Korea; 8 Department of Emergency Medicine, Uijeongbu St. Mary’s Hospital, The Catholic University of Korea College of Medicine, Uijeongbu-si, Korea; 9 Department of Emergency Medicine, Chonnam National University Hospital, Gwangju, Korea

**Keywords:** Out-of-hospital cardiac arrest, Neuron-specific enolase, Shockable rhythm, Non-shockable rhythm, Prognosis

## Abstract

**Background:**

Serum neuron-specific enolase (NSE) is the only recommended biomarker for multimodal prognostication in postcardiac arrest patients, but low sensitivity of absolute NSE threshold limits its utility. This study aimed to evaluate the prognostic performance of serum NSE for poor neurologic outcome in out-of-hospital cardiac arrest (OHCA) survivors based on their initial rhythm and to determine the NSE cutoff values with false positive rate (FPR) < 1% for each group.

**Methods:**

This study included OHCA survivors who received targeted temperature management (TTM) and had serum NSE levels measured at 48 h after return of spontaneous circulation in the Korean Hypothermia Network, a prospective multicenter registry from 22 university-affiliated teaching hospitals in South Korea between October 2015 and December 2018. The primary outcome was poor outcome at 6 month, defined as a cerebral performance category of 3–5.

**Results:**

Of 623 patients who underwent TTM with NSE measured 48 h after the return of spontaneous circulation, 245 had an initial shockable rhythm. Median NSE level was significantly higher in the non-shockable group than in the shockable group (104.6 [40.6–228.4] vs. 25.9 [16.7–53.4] ng/mL, *P* < 0.001). Prognostic performance of NSE assessed by area under the receiver operating characteristic curve to predict poor outcome was significantly higher in the non-shockable group than in the shockable group (0.92 vs 0.86). NSE cutoff values with an FPR < 1% in the non-shockable and shockable groups were 69.3 ng/mL (sensitivity of 76.0%) and 102.7 ng/mL (sensitivity of 42.1%), respectively.

**Conclusion:**

NSE prognostic performance and its cutoff values with FPR < 1% for predicting poor outcome in OHCA survivors who underwent TTM differed between shockable and non-shockable rhythms, suggesting postcardiac arrest survivor heterogeneity.

**Trial registration** KORHN-PRO, NCT02827422. Registered 11 September 2016—Retrospectively registered, https://clinicaltrials.gov/ct2/show/NCT02827422

**Supplementary Information:**

The online version contains supplementary material available at 10.1186/s13054-023-04603-6.

## Background

Blood biomarkers are an attractive modality for predicting outcomes in postcardiac arrest survivors, which are easy to obtain, quantifiable, observer independent, and insensitive to sedation [1–6]. Among the biomarkers, neuron-specific enolase (NSE) is the only recommended biomarker for multimodal prognostication in postcardiac arrest survivors [7, 8]. Current American Heart Association guidelines suggest that high NSE values within 72 h after cardiac arrest could be a robust predictor of poor outcome. The recently updated European Resuscitation Council/European Society of Intensive Care Medicine (ERC/ESICM) guidelines recommend that an NSE threshold level > 60 ng/mL at 48 and/or 72 h after cardiac arrest indicates poor outcomes in their prognostication strategy algorithm [7, 8]. However, varying NSE thresholds have been reported from different studies, and high NSE cutoff values for achieving high specificity result in low sensitivity [9, 10]. The clinical utility of the absolute NSE threshold is limited and further evidence is needed.

Serum NSE levels reflect neuronal cell damage and hypoxic-ischemic brain injury [11]; however, outliers have been reported in patients with good outcomes despite high NSE levels and those with poor outcomes despite low NSE levels [4, 7, 12]. In addition to extracerebral sources of NSE and the different measurement techniques, such inconsistency in NSE thresholds suggests that NSE level may be influenced by other clinical factors [4, 7, 12]. A recent study discovered the prognostic accuracy of NSE was decreased in elderly patients and those with a short time from collapse to return of spontaneous circulation (ROSC) (1–13 min) [12]. However, the impact of the initial rhythm on NSE prognostic value is unclear.

We hypothesized that optimal cutoff values of NSE for poor neurologic outcomes differ between shockable and non-shockable out-of-hospital cardiac arrest (OHCA). We aimed to assess the prognostic performance of NSE measured at 48 h after ROSC for poor neurologic outcome in comatose postcardiac arrest survivors treated with targeted temperature management (TTM) based on shockable and non-shockable initial rhythm and to establish NSE cutoff values for poor neurologic outcome with false positive rate (FPR) < 1% in shockable and non-shockable cardiac arrest, respectively.

## Methods

### Study design and population

This study is a sub-study of the Korean Hypothermia Network prospective registry (KORNH-PRO) [13]. Briefly, KORHN-PRO 1.0 is a web-based registry of OHCA survivors treated with TTM in 22 academic hospitals in South Korea between November 2015 and December 2018. KORHN-PRO registry (http://pro.korhn.or.kr/) collected 136 variables with 839 datasets and was registered on the International Clinical Trials Registry Platform (NCT02827422). All the variables were defined based on the Utstein template [14]. The participating institutions were distributed evenly across the country. The inclusion criteria of the KORNH-PRO registry were comatose adult (aged > 18 years) patients with non-traumatic OHCA who were treated with TTM, and the exclusion criteria were patients with active intracranial bleeding or acute ischemic stroke, limitations in therapy, a do-not-attempt resuscitation order, cerebral performance category (CPC) 3 or 4 before OHCA, body temperature < 30 °C on admission, and unknown outcomes for 6 months after the ROSC. Among the patients enrolled in the KORHN-PRO registry, this study included those who underwent serum NSE level measurement 48 h after ROSC.

All the enrolled patients received post-resuscitation care in accordance with the then-current international guidelines. The active withdrawal of life-sustaining treatment (WSLT) was legally prohibited in South Korea until February 2018, and all patients were admitted to hospitals with conservative treatment until death or recovery. The researchers conducted clinical follow-ups to determine the neurologic status according to the CPC score at discharge, 1 and 6 months after OHCA, through face-to-face visits or standardized follow-up telephone interviews with the patient or a primary caregiver (family member). The Institutional Review Board of all participating hospitals reviewed and approved the study protocol, including the Institutional Review Board of Asan Medical Center (2015–1052). Written informed consent was obtained from legal surrogates before the enrollment due to the patient’s comatose state.

### Data collection and endpoints

Demographic and clinical data were extracted from the web-based registry. The variables investigated in this study were: age, sex, medical history, witnessed arrest, bystander cardiopulmonary resuscitation (CPR), arrest cause, initial documented rhythm (shockable vs. non-shockable), time from CPR to ROSC, time from ROSC to TTM initiation, target temperature, serum NSE levels measured 48 h after ROSC, and the poor neurological outcomes at one and six months after cardiac arrest, defined as a CPC score of 3–5. Of the 22 sites, 17 provided NSE levels 48 h after ROSC.

### Statistical analyses

Continuous variables were presented as median with interquartile ranges due to their non-normal distribution based on the Kolmogorov–Smirnov test. Categorical variables were expressed as numbers and percentages. Demographic and clinical characteristics between groups with initial shockable and non-shockable rhythms were compared using the chi-square or Fisher’s exact test for categorical variables and the Mann–Whitney U test for continuous variables, as appropriate. Univariate logistic analysis was first performed to evaluate the prognostic ability of each variable, and the variables with a *P* value of < 0.05 in the univariate analysis were analyzed by multivariate logistic regression based on a backward elimination method. The results of the logistic regression analysis were summarized using odds ratios (ORs) and the respective 95% confidence intervals (CIs). Variables were tested for goodness of fit using a Hosmer–Lemeshow test. The receiver operating characteristic curves with 95% confidence intervals were examined to determine the performance of the NSE level 48 h after ROSC in predicting a poor neurological outcome at six months. The area under the curve (AUC) for each group was calculated and compared using DeLong’s test [15]. The optimal cutoff value of the NSE level at 48 h for each group was determined using the Youden index, which defines the cutoff in terms of the maximal sum of sensitivity and specificity. We also determined the NSE cutoff values with FPR < 1% in shockable and non-shockable cardiac arrest. A two-tailed *P* value of < 0.05 was considered significant. Statistical analyses were conducted using SPSS 24.0 (SPSS Inc., Chicago, IL, USA) and MedCalc® Statistical Software version 20.118 (MedCalc Software Ltd, Ostend, Belgium; https://www.medcalc.org; 2022).

## Results

**Fig. 1:**
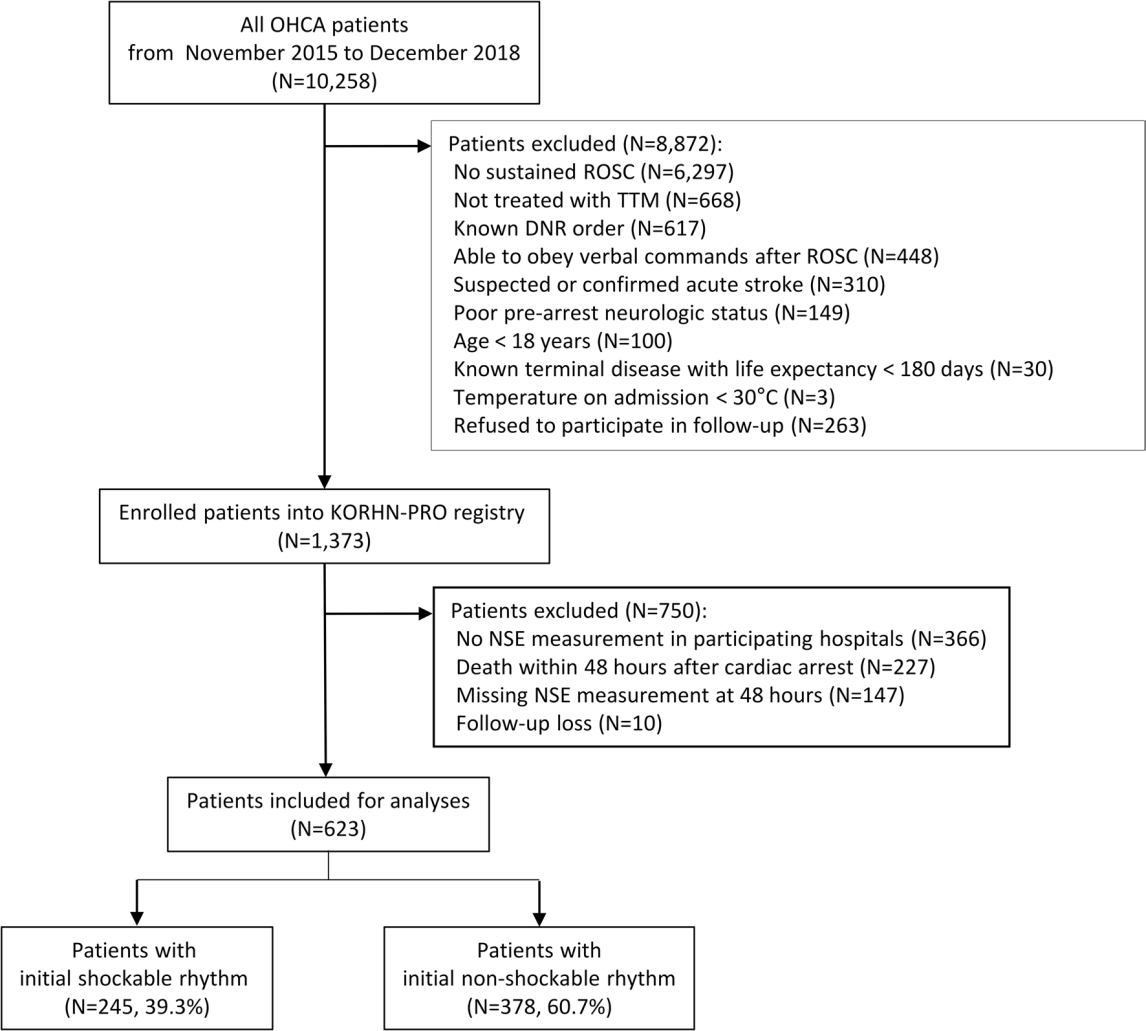
Flow diagram of the patient selection process. DNR, do-not-attempt resuscitation; KORHN-PRO, Korean Hypothermia Network prospective registry; OHCA, out-of-hospital cardiac arrest; ROSC, return of spontaneous circulation; TTM, targeted temperature management; NSE, neuron-specific enolase

During the study period, 1373 comatose OHCA survivors were enrolled in the KORHN-PRO registry (Fig. 1). Among these cases, 623 patients were included in our study after excluding 750 patients who did not undergo the NSE test in participating hospitals during post-resuscitation care (*n* = 366), died within 48 h after cardiac arrest (*n* = 227), were lost to measured NSE level at 48 h (*n* = 147), or were not followed up (*n* = 10).

The demographic and clinical characteristics of the patients with initial shockable and non-shockable rhythms are summarized in Table 1. Patients with initial shockable rhythm were younger. Patients with initial shockable rhythm had a higher rate of witnessed cardiac arrest (83.2% vs. 60.9%, *P* < 0.001) and received bystander CPR more frequently (71.0% vs. 56.9%, *P* < 0.001). The cause of arrest also differed between both groups; almost all patients with initial shockable rhythm were presumed to have cardiogenic cause of cardiac arrest (96.7%), approximately two-fifths (41.8%) of those with initial non-shockable rhythm. Resuscitation duration was significantly shorter in the initial shockable group, whereas the no-flow time did not differ between both groups. Serum NSE level at 48 h after ROSC was lower in the patients with initial shockable rhythm (median, vs. 104.55 ng/mL, *P* < 0.001). Time interval to TTM and target temperature did not differ between the two groups. However, withholding or withdrawing therapies occurred more frequently in patients in the initial non-shockable group. The median time to withdrawing therapies from ROSC was 121.0 h in this cohort. The rate of good neurological outcome at 6 months was significantly higher in the initial shockable rhythm group than in the non-shockable group (69.0% vs. 16.1%, *P* < 0.001).

**Table 1:** Demographic and clinical characteristics of study patients according to initial documented cardiac arrest rhythm

Characteristics	Total (*N* = 623)	Patients with initial shockable rhythm (*N* = 245)	Patients with initial non-shockable rhythm (*N* = 378)	p value
Age, years	58.0 (47.0–68.0)	56.0 (44.5–63.0)	60.0 (48.0–71.0)	< 0.001
Male	455 (73.0%)	197 (80.4%)	258 (68.3%)	0.001
Previous medical history				
Hypertension	221 (35.5%)	78 (31.8%)	143 (37.8%)	0.13
Diabetes mellitus	146 (23.4%)	33 (13.5%)	113 (29.9%)	< 0.001
Acute myocardial infarction	41 (6.6%)	18 (7.3%)	23 (6.1%)	0.54
Congestive heart failure	21 (3.4%)	7 (2.9%)	14 (3.7%)	0.57
Chronic kidney disease	43 (6.9%)	3 (1.6%)	39 (10.3%)	< 0.001
Witnessed	429 (69.8%)	203 (83.2%)	226 (60.9%)	< 0.001
Bystander CPR	389 (62.4%)	174 (71.0%)	215 (56.9%)	< 0.001
Arrest cause				< 0.001
Presumed cardiac	395 (63.4%)	237 (96.7%)	158 (41.8%)	
Other medical cause	96 (15.4%)	5 (2.0%)	91 (24.1%)	
External cause	132 (21.2%)	3 (1.2%)	129 (34.1%)	
No flow time, min	1.0 (0.0–7.0)	1.0 (0.0–5.0)	1.0 (0.0–8.0)	0.14
Resuscitation duration, min	23.0 (13.0–36.0)	19.0 (11.0–32.5)	25.0 (15.0–37.0)	0.001
Time from ROSC to TTM initiation, hours	3.6 (2.5–5.1)	3.6 (2.5–4.9)	3.6 (2.4–5.3)	0.46
Target temperature				0.45
33 °C	450 (72.2%)	181 (73.9%)	269 (71.2%)	
34–35 °C	137 (22.0%)	48 (19.6%)	89 (23.5%)	
36 °C	36 (5.8%)	16 (6.5%)	20 (5.3%)	
ECMO < 48 h	29 (4.7%)	19 (7.8%)	10 (2.6%)	0.003
Hemodialysis < 48 h	104 (16.7%)	28 (11.4%)	76 (20.1%)	0.005
Intra-arterial balloon pump	1 (0.2%)	1 (0.4%)	0 (0%)	0.39
NSE at 48 h after ROSC, ng/mL	50.02 (22.58–182.07)	25.89 (16.69–53.40)	104.55 (40.63–228.38)	< 0.001
Absent pupillary reflex at ≥ 72 h*	243 (39.0%)	37 (15.1%)	206 (54.5%)	< 0.001
Withholding or withdrawing therapies				< 0.001
None	522 (83.8%)	228 (93.1%)	294 (77.8%)	
No therapeutic escalation	14 (2.2%)	0 (0%)	14 (3.7%)	
No CPR in case of a re-arrest	70 (11.2%)	14 (5.7%)	56 (14.8%)	
Withdrawing therapies	17 (2.7%)	3 (1.2%)	14 (3.7%)	
Time from ROSC to withdrawing therapies, hours	121.0 (91.0–198.0), *n* = 17	94.0 (84.0–96.5), *n* = 3	160.0 (114.0–201.0), *n* = 14	0.12
Good neurologic outcome at 6 month	230 (36.9%)	169 (69.0%)	61 (16.1%)	< 0.001

Values are expressed as median (interquartile ranges) or *n* (%) as appropriate

*The results were missing in 41 patients, including 17 in the shockable rhythm group (6.9%) and 24 in the non-shockable rhythm group (6.3%), respectively

*CPR* Cardiopulmonary resuscitation; *ECMO* Extracorporeal membrane oxygenation; *ROSC* Return of spontaneous circulation; *TTM* Targeted temperature management; *NSE* Neuron-specific enolase

In multivariate logistic regression analysis using the significant variables in the univariate logistic regression analysis, NSE level at 48 h after ROSC (adjusted OR, 1.05; 95% CI, 1.04–1.06; *P* < 0.001) was associated with poor neurologic outcomes at six months (Table 2). In subgroup analysis according to initial documented arrest rhythm (shockable or non-shockable), the adjusted ORs of NSE at 48 h after ROSC were 1.05 (95% CI, 1.03–1.06; *P* < 0.001) in patients with shockable rhythm and 1.06 (95% CI, 1.04–1.09; *P* < 0.001) in patients with non-shockable rhythm, respectively.

**Table 2:** Logistic regression analysis for poor neurologic outcome at six months in the patients with out-of-hospital cardiac arrest

Characteristics	OR	95% CI	P value	Adjusted OR	95% CI	P value
Age, years	1.03	1.02–1.04	< 0.001	1.05	1.02–1.07	< 0.001
Male	0.65	0.44–0.95	0.03			
Previous medical history						
Hypertension	1.52	1.07–2.15	0.02			
Diabetes mellitus	2.30	1.50–3.51	< 0.001	1.74	0.91–3.33	0.09
Acute myocardial infarction	1.14	0.58–2.22	0.70			
Congestive heart failure	1.18	0.47–2.96	0.73			
Chronic kidney disease	2.33	1.10–4.94	0.03			
Arrest characteristics						
Witnessed	0.39	0.27–0.58	< 0.001			
Bystander CPR	0.63	0.45–0.89	0.01			
Initial shockable rhythm	0.09	0.06–0.13	< 0.001	0.22	0.12–0.41	< 0.001
Resuscitation duration, min	1.06	1.04–1.07	< 0.001	1.02	1.00–1.04	0.02
Arrest cause						
External cause	Reference		< 0.001	Reference		0.03
Presumed cardiac cause	0.06	0.19–0.19	< 0.001	0.33	0.13–0.83	0.02
Other medical cause	0.77	0.33–1.76	0.53	0.74	0.24–2.25	0.59
Target temperature						
33 °C	Reference		0.58			
34–35 °C	1.19	0.80–1.78	0.40			
36 °C	0.84	0.42–4.58	1.68			
NSE at 48 h after ROSC, ng/mL	1.05	1.04–1.06	< 0.001	1.05	1.04–1.06	< 0.001

*CI* Confidence interval; *CPC* Cerebral Performance Category; *CPR* Cardiopulmonary resuscitation; *NSE* Neuron-specific enolase; *OR* Odds ratio

**Fig. 2:**
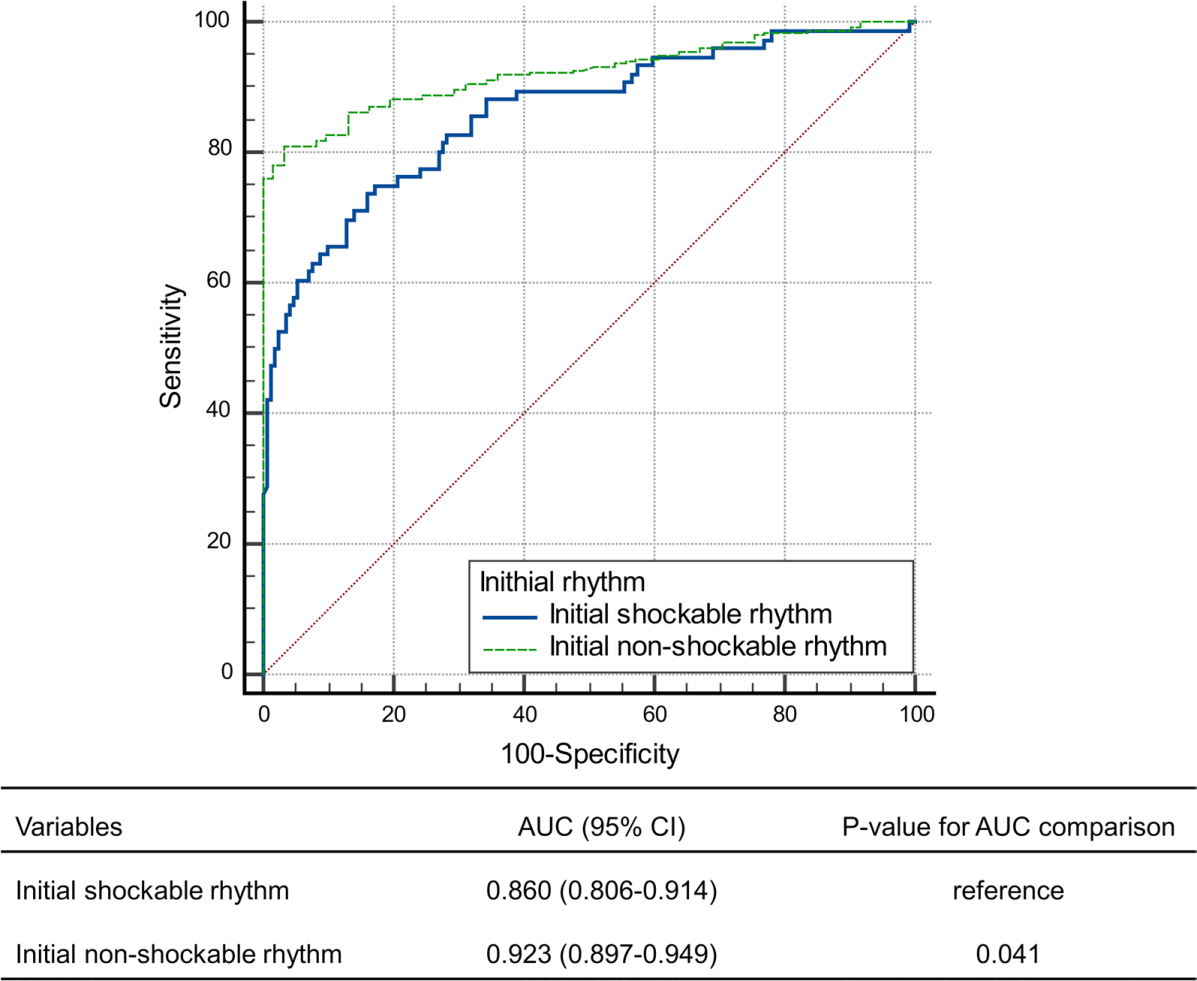
Prognostic performance of neuron-specific enolase in out-of-hospital cardiac arrest treated with targeted temperature management stratified by initial shockable and non-shockable rhythms for predicting a poor neurological outcome at 6 months. AUC, area under the curve; CI, confidence interval

The predictive performance of NSE level at 48 h after ROSC for poor neurologic outcome at 6 months was superior in patients with initial non-shockable rhythm (AUC, 0.923; 95% CI, 0.897–0.949) to those with initial shockable rhythm (AUC, 0.860; 95% CI, 0.806–0.914, *P* = 0.04) (Fig. 2). The optimal cutoff value based on the Youden index was 57.61 ng/mL in the total population; 35.34 ng/mL and 49.19 ng/mL in patients with initial shockable rhythm and non-shockable rhythm, respectively (Table 3). An NSE value of > 102.72 ng/mL in the initial shockable group predicted a poor neurological outcome at 6 months with a 0.6% FPR and a 42.1% sensitivity. In the initial non-shockable group, the NSE cutoff value with an FPR < 1% was 69.28 ng/mL with a 76.0% sensitivity. When the previously suggested NSE value of > 60 ng/mL was applied, it predicted a poor outcome with a 4.7% FPR and a 57.9% sensitivity in the initial shockable group and a 3.3% FPR and a 78.2% sensitivity in the initial non-shockable group.

**Table 3:** Predictive value of neuron-specific enolase levels 48 h after the return of spontaneous circulation for poor neurologic outcome

Timing	Patients	AUC	Cutoff value (ng/mL) (Classification)	Sensitivity (95% CI)	Specificity (95% CI)	PPV (95% CI)	NPV (95% CI)	Accuracy (95% CI)
1-month	Total (*N* = 623)	0.91 (0.89–0.93)	57.61 (Youden index)	74.5 (69.9–87.7)	94.7 (91.0–97.2)	96.1 (93.4–97.7)	68.0 (64.2–71.6)	81.9 (78.6–84.8)
			86.95 (< 1% FPR)	63.1 (58.2–67.9)	99.1 (96.9–99.9)	99.2 (96.9–99.8)	60.7 (57.5–63.7)	76.2 (72.7–79.5)
			60 (Previously suggested)	73.5 (68.9–77.8)	95.2 (91.5–97.6)	93.4 (93.7–97.9)	67.3 (63.5–70.9)	81.4 (78.1–84.4)
	Shockable rhythm (*N* = 245)	0.85 (0.80–0.91)	34.15 (Youden index)	72.5 (61.4–81.9)	83.0 (76.4–88.4)	67.4 (59.0–74.9)	86.2 (81.3–90.0)	79.6 (74.0–84.5)
			102.72 (< 1% FPR)	40.0 (29.2–51.6)	99.4 (96.7–100.0)	97.0 (81.7–99.6)	77.4 (74.1–80.3)	80.0 (74.4–84.8)
			60 (Previously suggested)	53.8 (42.2–65.0)	94.6 (89.9–97.5)	82.7 (71.0–90.3)	80.8 (76.9–84.3)	81.2 (75.8–85.9)
	Non-shockable rhythm (*N* = 378)	0.92 (0.90–0.95)	49.19 (Youden index)	81.3 (76.6–85.5)	96.8 (88.8–99.6)	99.2 (97.0–99.8)	50.4 (44.6–56.3)	83.9 (79.8–87.4)
			69.28 (< 1% FPR)	76.3 (71.2–80.9)	100.0 (94.2–100.0)	100	45.3 (40.4–50.2)	80.2 (75.8–84.1)
			60 (Previously suggested)	78.5 (73.5–82.9)	96.8 (88.8–99.6)	99.2 (97.0–99.8)	46.9 (41.6–52.3)	81.7 (77.4–85.5)
6-month	Total (*N* = 623)	0.91 (0.89–0.93)	57.32 (Youden index)	75.3 (70.8–79.5)	95.2 (91.6–97.6)	96.4 (93.8–98.0)	69.3 (65.6–72.9)	82.7 (79.5–85.6)
			86.95 (< 1% FPR)	61.5 (56.3–66.4)	99.2 (97.2–99.9)	99.1 (96.6–99.8)	63.6 (60.6–66.5)	76.7 (73.2–80.0)
			60 (Previously suggested)	74.3 (69.7–78.6)	95.7 (92.2–97.9)	96.7 (94.1–98.2)	68.5 (64.8–72.1)	82.2 (79.0–85.1)
	Shockable rhythm (*N* = 245)	0.86 (0.81–0.91)	35.34 (Youden index)	73.7 (62.3–83.1)	84.0 (77.6–89.2)	67.5 (58.9–75.0)	87.7 (82.9–91.2)	80.8 (75.3–85.6)
			102.72 (< 1% FPR)	42.1 (30.9–54.0)	99.4 (96.8–100.0)	97.0 (81.7–99.6)	79.3 (75.9–82.2)	81.6 (76.2–86.3)
			60 (Previously suggested)	57.9 (46.0–69.1)	95.3 (90.9–97.9)	84.6 (73.1–91.7)	83.4 (79.4–86.8)	83.7 (78.4–88.1)
	Non-shockable rhythm (*N* = 378)	0.92 (0.90–0.95)	49.19 (Youden index)	81.1 (76.3–85.2)	96.7 (88.7–99.6)	99.2 (97.1–99.8)	49.6 (43.8–55.4)	83.6 (79.5–87.2)
			69.28 (< 1% FPR)	76.0 (70.9–80.6)	100.0 (94.1–100.0)	100	44.5 (39.8–49.4)	79.9 (75.5–83.8)
			60 (Previously suggested)	78.2 (73.3–82.7)	96.7 (88.7–99.6)	99.2 (96.9–99.8)	46.1 (40.9–51.4)	81.2 (76.9–85.0)

AUC, area under the curve; CI, confidence interval; FPR, false positive rate; NPV, negative predictive value; PPV, positive predictive value

## Discussion

In this prospective multicenter registry-based cohort study, we discovered that NSE level measured at 48 h revealed better prognostic performance for poor neurological outcome in patients with initial non-shockable rhythm than in those with initial shockable rhythm (AUC, 0.92 vs. 0.86, *P* = 0.04). We also identified different NSE cutoff values with an FPR < 1% for the shockable and non-shockable rhythm groups as 103 ng/mL and 69 ng/mL, respectively. Our findings suggest the heterogeneity of postcardiac arrest survivors and implied that interpretation of some diagnostic test such as NSE may need to vary depending on the patient’s important prognostic characteristics, such as initial rhythm.

Previous guidelines suggested an NSE threshold of > 33 ng/mL on days 1–3 for poor outcomes with FPR < 3% [16]. However, patients with high NSE levels sometimes had good neurologic outcomes, particularly patients treated with TTM [17]. The results are conflicting; however, higher NSE thresholds have been warranted to avoid the wrong prognostic decision-making. In the TTM substudy, NSE had an AUC of 0.85–0.86 at 48–72 h, and NSE cutoff values with FPR from 5 to 1% ranged from 42 to 68 ng/mL, and 33–45 ng/mL at 48 and 72 h, respectively [9]. A multicenter study on 1,053 in-hospital cardiac arrest and OHCA patients treated with TTM at 32–34 °C reported that the NSE at 72 h had an AUC of 0.85–0.90 and the NSE threshold for poor outcome with FPR 0.5% was > 90 μg/L [17]. These are consistent with our result that the prognostic value of NSE at 48 h for poor outcome was an AUC of 0.91 (95% CI, 0.89–0.93). When postcardiac arrest survivors were classified according to their initial rhythm, NSE at 48 h in initial non-shockable rhythm group had the highest AUC of 0.92 (95% CI 0.90–0.95).

A higher prognostic value of NSE in initial non-shockable rhythm group than initial shockable rhythm group may be attributed to the following factors. Firstly, a non-cerebral cause-of-death caused by circulatory failure would be more prevalent in OHCA patients with initial shockable rhythm compared to the patients with non-shockable rhythm, where death caused by neurologic injury may be more prevalent [18–20]. Additionally, the predictive performance of NSE was positively associated with the severity of hypoxic-ischemic damage. A recent study demonstrated that the prognostic performance of NSE differed by age and resuscitation duration [12]. The predictive value was poor for the patients with short resuscitation duration (AUC of 0.45) but was good for those with long resuscitation duration (AUC of 0.84). The patients with initial non-shockable rhythm in our study appeared to have more severe hypoxic-ischemic damage including brain, which the patients with initial non-shockable rhythm showed prolonged resuscitation duration, higher NSE level at 48 h after ROSC and higher rate of absent pupillary reflex at ≥ 72 h after ROSC compared to those with shockable rhythm. A significant proportion of OHCA patients died due to protracted shock and multiorgan failure early after cardiac arrest before any possible neurological outcome assessment, which indicates the severity of hypoxic-ischemic damage [21]. In our study, 227 of 1373 (16.5%) patients died within 48 h, and the rate of initial non-shockable rhythm was significantly higher in excluded patients with early death compared to study patients (see additional file 1, 81% vs. 61%; *P* < 0.001). Mortality from post-cardiac arrest shock and brain injury share similar risk factors [18], and the extracerebral organ failure occurring later did not significantly influence the outcome [21]. NSE serves as a surrogate marker for brain injury; thus, the predictive value of NSE is limited in other medical conditions resulting in deterioration. Thirdly, the occurrence of death after awakening as the result of persistent circulatory failure or cardiac ischemia might occur more frequently in the patients with initial shockable rhythm which contribute to decrease the predictive performance of NSE [22]. Finally, NSE level increases in several medical conditions, such as neuroendocrine tumors, small cell lung cancer, and use of medical devices, which potentially cause hemolysis, including extracorporeal membrane oxygenation, hemodialysis, and intra-arterial balloon pump, and can lead to false positives. In our study, extracorporeal membrane oxygenation was performed more frequently in patients with shockable rhythm, whereas hemodialysis was more frequently in those with non-shockable rhythm.

The 2021 ERC/ESICM guidelines modified the NSE recommendation to a 60 ng/mL cutoff level at 48 h and/or 72 h, given the prognostication algorithm for post-resuscitation care. A recent validation study reported an FPR of 4% for poor outcome prediction at the guideline-recommended NSE threshold of > 60 ng/mL and revealed that a < 2% FPR for poor neurologic outcome prediction was achieved with an NSE cutoff value of > 101 ng/mL at 48 h [23]. In our cohort, the guideline-recommended NSE cutoff value of > 60 ng/mL at 48 h yielded a specificity of 95.7%, an FPR of 4.3%, and a sensitivity of 74.3% for predicting poor neurologic outcomes at 6 months. The threshold was increased to 86.95 ng/mL with an FPR of 1% and a sensitivity of 61.5%. Stammet et al. reported that FPR decreased from 5 to 1% and increased the NSE threshold from 42 to 68 ng/mL with decreasing sensitivity from 61 to 47% [9]. When the threshold was set to achieve an FPR of 0%, an impractically high NSE threshold, the sensitivity decreased to 27% [6, 9]. To enhance the clinical utility of NSE, establishing different thresholds for different patient populations would be warranted.

Current guidelines for post-resuscitation care for OHCA survivors do not differentiate the OHCA patients based on their initial rhythm. However, some experts suggested that different treatment strategies for each group should be considered [24, 25]. When using the NSE level as a prognostic marker for OHCA survivors, stratifying OHCA survivors based on the initial rhythm may be a reasonable approach. In our study, we identified different NSE cutoff values with an FPR < 1% for the shockable and non-shockable rhythm groups as 103 ng/mL with a 42.1% sensitivity and 69 ng/mL with a 76.0% sensitivity for predicting a poor neurological outcome at 6 months. These NSE thresholds are higher than the guideline-recommended NSE threshold of > 60 ng/mL, particularly in shockable rhythm patients. Our findings suggest that some postcardiac arrest patients with initially shockable rhythm with higher NSE levels > 60 ng/mL may have a chance of good neurologic outcome. A multimodal approach for predicting neurological outcomes is essential to avoid unethical decisions for WLST. Moreover, our findings implied that physicians consider to continue postcardiac arrest care and re-evaluate patients, particularly those presenting with a shockable rhythm, although they showed NSE > 60 ng/mL at 48 h and/or 72 h, along with one of the poor prognostic factors including no pupillary and corneal reflexes at ≥ 72 h, bilaterally absent N20 somatosensory evoked potentials wave at ≥ 24 h, highly malignant electroencephalography at > 24 h, status myoclonus ≤ 72 h or a diffuse and extensive anoxic injury on brain image.

Our study had several strengths. First, our findings were less biased as only a small percentage (2.7%) of our study participants received WLST due to the legal prohibition in South Korea until February 2018. Second, our study included 623 Asian patients from a nationwide, multicenter prospective registry, which adds to the generalizability of our findings beyond the Western populations that have been previously studied [9, 17]. Despite these strengths, our study had several limitations. First, excluding 513 out of 1,373 (37.4%) patients who did not have NSE data at 48 h may have contributed to a selection bias. Nevertheless, comparing the clinical characteristics and neurologic outcomes between the study patients and those excluded from our study due to a lack of NSE values at 48 h (see Additional file 2) revealed no significant differences. However, the excluded patients who died < 48 h after cardiac arrest were older and had longer resuscitation duration compared to the study patients (see Additional file 1). Second, we included the patients who underwent renal replacement therapy and extracorporeal membrane oxygenation, which could have led to outlier NSE values. The participating hospitals are academic hospitals with a department of laboratory medicine, and the laboratory medicine specialist reported NSE values after assessing the hemolysis index. However, the hemolysis index of each sample was not included separately, and thus, the impact of NSE hemolysis on prognostic performance should be acknowledged. Third, NSE thresholds would be associated with poor outcome, but it is notable that the patients with an initial non-shockable rhythm appeared to be more critically ill compared to those with an initial shockable rhythm. We identified significantly different characteristics between the two groups, which could potentially be used as predictive factors for outcomes, such as age, witnessed cardiac arrest, arrest cause and resuscitation duration. These disparities might lead to significantly different NSE cutoff values. Fourth, we did not differentiate the patients who died after neurologic recovery and did not evaluate the cause of death, which may have led to survivorship bias. Fifth, two different NSE measurement instruments were utilized though the participating centers [26]. The Roche-method (NSE Cobas e601, CNSE) resulted in average 15% higher values of NSE compared to the DiaSorin-method (LIAISON®NSE, LNSE) [27]. Finally, the inherent risks and potential biases of the registry-based study should be acknowledged. Although the central manager of KORNH-PRO registry regularly qualified the data, there may be instances of incomplete or missing data, non-standardized treatment across all participating centers in the registry and selection bias.

## Conclusions

NSE value at 48 h for predicting poor outcomes in OHCA survivors treated with TTM revealed superior predictive performance in those with initial non-shockable rhythm than those with an initial shockable rhythm. The cutoff values with an FPR < 1% were much higher in patients with initial shockable rhythm than the previously suggested NSE cutoff of 60 ng/mL and in patients with initial non-shockable rhythm. Our findings suggested the heterogeneity of postcardiac arrest survivors and some important arrest characteristics such as initial rhythm could be used to improve the predictive performance of NSE. However, it is essential to acknowledge that our study serves as a starting point for further investigations rather than providing conclusive evidence. Further validation studies, ideally randomized controlled trials, are warranted to evaluate whether the utilization of different NSE cutoff values can improve the performance of the post-cardiac arrest prognostication strategy algorithm.

## Supplementary Information

**Additional file 1.** Demographic and clinical characteristics of the study patients and excluded patients who died within 48 hours after cardiac arrest

**Additional file 2.** Demographic and clinical characteristics of patients with out-of-hospital cardiac arrest treated with targeted temperature management with and without neuron-specific enolase level measurement at 48 h

## Data Availability

The datasets used and/or analyzed during the current study are available from the corresponding author on reasonable request.

## Abbreviations

AUC: Area under the curve
CI: Confidence interval
CPR: Cardiopulmonary resuscitation
ERC/ESICM: European Resuscitation Council/European Society of Intensive Care Medicine
FPR: False positive rate
KORHN-PRO: Korean Hypothermia Network prospective registry
NSE: Neuron-specific enolase
OHCA: Out-of-hospital cardiac arrest
ROSC: Return of spontaneous circulation
TTM: Targeted temperature management
WLST: Withdrawal of life-sustaining treatment
