# Correction to: Different neuroprognostication thresholds of neuron-specific enolase in shockable and non-shockable out-of-hospital cardiac arrest: a prospective multicenter observational study in Korea (the KORHN-PRO registry)

**DOI:** 10.1186/s13054-025-05252-7

**Published:** 2025-01-17

**Authors:** Youn‑Jung Kim, Yong Hwan Kim, Chun Song Youn, In Soo Cho, Su Jin Kim, Jung Hee Wee, Yoo Seok Park, Joo Suk Oh, Byung Kook Lee, Won Young Kim

**Affiliations:** 1 Department of Emergency Medicine, Asan Medical Center, University of Ulsan College of Medicine, 88, Olympic‑ro 43‑gil, Songpa‑gu, Seoul, 05505 Korea; 2 Departments of Emergency Medicine, Samsung Changwon Hospital, Sungkyunkwan University School of Medicine, Changwon, Korea; 3 Department of Emergency Medicine, Seoul St. Mary’s Hospital, College of Medicine, The Catholic University of Korea, Seoul, Korea; 4 Department of Emergency Medicine, Hanil General Hospital, Seoul, Korea; 5 Department of Emergency Medicine, Korea University College of Medicine, Seoul, Korea; 6 Department of Emergency Medicine, Yeouido St. Mary’s Hospital, The Catholic University of Korea College of Medicine, Seoul, Korea; 7 Department of Emergency Medicine, Yonsei University College of Medicine, Seoul, Korea; 8 Department of Emergency Medicine, Uijeongbu St. Mary’s Hospital, The Catholic University of Korea College of Medicine, Uijeongbu‑si, Korea; 9 Department of Emergency Medicine, Chonnam National University Hospital, Gwangju, Korea

**Correction: Crit Care 27, 313 (2023)** 10.1186/s13054-023-04603-6

Following publication of the original article [1], the authors identified an error in Results section in the Abstract.

The incorrect sentence in the Results section in the Abstract reads:

NSE cutoff values with an FPR < 1% in the non-shockable and shockable groups were 69.3 (sensitivity of 42.1%) and 102.7 ng/mL (sensitivity of 76%), respectively.

The correct sentence in the Results section within the Abstract should read:

NSE cutoff values with an FPR < 1% in the non-shockable and shockable groups were 69.3 ng/mL (sensitivity of 76.0%) and 102.7 ng/mL (sensitivity of 42.1%), respectively.

The given sentence in the Abstract has been updated above and the original article [1] has been corrected.
