# Supplementary material for: Different neuroprognostication thresholds of neuron-specific enolase in shockable and non-shockable out-of-hospital cardiac arrest: a prospective multicenter observational study in Korea (the KORHN-PRO registry)
Source: Crit Care. 2023 Aug 9;27:313. doi: 10.1186/s13054-023-04603-6 (PMC10413805; doi:10.1186/s13054-023-04603-6)
Supplement: Supplementary file 1 — Additional file 1. Demographic and clinical characteristics of the study patients and excluded patients who died within 48 hours after cardiac arrest [file 13054_2023_4603_MOESM1_ESM.docx]

**Additional file 1.** Demographic and clinical characteristics of the study patients and excluded patients who died within 48 hours after cardiac arrest

| Characteristics | Patients with NSE level at 48 hours (N=623) | Patients who died < 48 hours after cardiac arrest (N=227) | P-value |
| --- | --- | --- | --- |
| Age, years | 58.0 (47.0–68.0) | 68.0 (54.0–76.0) | <0.001 |
| Male | 455 (73.0%) | 164 (72.2%) | 0.82 |
| Previous medical history |  |  |  |
| Hypertension | 221 (35.5%) | 94 (41.4%) | 0.11 |
| Diabetes mellitus | 146 (23.4%) | 75 (33.0%) | 0.005 |
| Acute myocardial infarction | 41 (6.6%) | 14 (6.2%) | 0.83 |
| Congestive heart failure | 21 (3.4%) | 11 (4.8%) | 0.32 |
| Chronic kidney disease | 43 (6.9%) | 20 (8.8%) | 0.35 |
| Witnessed | 429 (69.8%) | 147 (65.3%) | 0.22 |
| Bystander CPR | 389 (62.4%) | 129 (56.8%) | 0.14 |
| Initial shockable rhythm | 245 (39.3%) | 44 (19.4%) | <0.001 |
| Arrest cause |  |  | 0.12 |
| Presumed cardiac | 395 (63.4%) | 138 (60.8%) |  |
| Other medical cause | 96 (15.4%) | 48 (21.1%) |  |
| External cause | 132 (21.2%) | 41 (18.1%) |  |
| No flow time, min | 1.0 (0.0–-7.0) | 2.0 (0.0–9.8) | 0.05 |
| Resuscitation duration, min | 23.0 (13.0–36.0) | 35.0 (24.3–48.0) | <0.001 |
| Withholding or withdrawing therapies |  |  | <0.001 |
| None | 522 (83.8%) | 117 (51.5%) |  |
| No therapeutic escalation | 14 (2.2%) | 8 (3.5%) |  |
| No CPR in case of a re-arrest | 70 (11.2%) | 100 (44.1%) |  |
| Withdrawing therapies | 17 (2.7%) | 2 (0.9%) |  |

Values are expressed as median (interquartile ranges) or n (%) as appropriate.

Abbreviations: CPR, cardiopulmonary resuscitation; ROSC, return of spontaneous circulation; TTM, targeted temperature management.
