# Supplementary material for: Different neuroprognostication thresholds of neuron-specific enolase in shockable and non-shockable out-of-hospital cardiac arrest: a prospective multicenter observational study in Korea (the KORHN-PRO registry)
Source: Crit Care. 2023 Aug 9;27:313. doi: 10.1186/s13054-023-04603-6 (PMC10413805; doi:10.1186/s13054-023-04603-6)
Supplement: Supplementary file 2 — Additional file 2. Demographic and clinical characteristics of patients with out-of-hospital cardiac arrest treated with targeted temperature management with and without neuron-specific enolase level measurement at 48 h [file 13054_2023_4603_MOESM2_ESM.docx]

**Additional file 2.** Demographic and clinical characteristics of patients with out-of-hospital cardiac arrest treated with targeted temperature management with and without neuron-specific enolase level measurement at 48 h

| Characteristics | Patients with NSE level at 48 hours (N=623) | Patients without NSE level at 48 hours (N=513) | P-value |
| --- | --- | --- | --- |
| Age, years | 58.0 (47.0–68.0) | 57.0 (46.0–68.0) | 0.33 |
| Male | 455 (73.0%) | 355 (69.2%) | 0.16 |
| Previous medical history |  |  |  |
| Hypertension | 221 (35.5%) | 168 (32.7%) | 0.34 |
| Diabetes mellitus | 146 (23.4%) | 101 (19.7%) | 0.13 |
| Acute myocardial infarction | 41 (6.6%) | 29 (5.7%) | 0.52 |
| Congestive heart failure | 21 (3.4%) | 14 (2.7%) | 0.53 |
| Chronic kidney disease | 43 (6.9%) | 39 (7.6%) | 0.65 |
| Witnessed | 429 (69.8%) | 369 (72.4%) | 0.34 |
| Bystander CPR | 389 (62.4%) | 325 (63.4%) | 0.75 |
| Arrest cause |  |  | 0.35 |
| Presumed cardiac | 395 (63.4%) | 317 (61.8%) |  |
| Other medical cause | 96 (15.4%) | 70 (13.6%) |  |
| External cause | 132 (21.2%) | 126 (24.6%) |  |
| No flow time, min | 1.0 (0.0–-7.0) | 1.0 (0.0–6.0) | 0.28 |
| Resuscitation duration, min | 23.0 (13.0–36.0) | 23.0 (13.0–34.0) | 0.81 |
| Good neurologic outcome at 1 month | 227 (36.4%) | 196 (38.2%) | 0.54 |
| Good neurologic outcome at 6 month | 230 (36.9%) | 195 (38.0%) | 0.71 |

Values are expressed as median (interquartile ranges) or n (%) as appropriate.

Abbreviations: CPR, cardiopulmonary resuscitation; ROSC, return of spontaneous circulation; TTM, targeted temperature management.
